# The oncogenic role of Epstein–Barr virus‐encoded microRNAs in Epstein–Barr virus‐associated gastric carcinoma

**DOI:** 10.1111/jcmm.13354

**Published:** 2017-10-09

**Authors:** Jinglin Zhang, Tingting Huang, Yuhang Zhou, Alfred S. L. Cheng, Jun Yu, Ka Fai To, Wei Kang

**Affiliations:** ^1^ Department of Anatomical and Cellular Pathology State Key Laboratory of Oncology in South China Prince of Wales Hospital The Chinese University of Hong Kong Hong Kong SAR China; ^2^ Institute of Digestive Disease Partner State Key Laboratory of Digestive Disease The Chinese University of Hong Kong Hong Kong SAR China; ^3^ Li Ka Shing Institute of Health Science Sir Y.K. Pao Cancer Center The Chinese University of Hong Kong Hong Kong SAR China; ^4^ Shenzhen Research Institute The Chinese University of Hong Kong Shenzhen China; ^5^ School of Biomedical Sciences The Chinese University of Hong Kong Hong Kong China; ^6^ Department of Medicine and Therapeutics The Chinese University of Hong Kong Hong Kong China

**Keywords:** gastric cancer, Epstein–Barr virus, microRNA

## Abstract

Epstein–Barr virus (EBV) infection is detected in various epithelial malignancies, such as nasopharyngeal carcinoma (NPC) and gastric cancer (GC). EBV comprises some unique molecular features and encodes viral genes and microRNAs (miRNAs) by its own DNA sequence. EBV genes are required to maintain latency and contribute to oncogenic property. miRNAs encoded by EBV have been shown to contribute to initiation and progression of EBV‐related malignancies. By a number of genomic profiling studies, some EBV miRNAs were confirmed to be highly expressed in EBV‐associated gastric cancer (EBVaGC) samples and cell lines. The majority host targets of the EBV miRNAs are important for promoting cell growth and inhibiting apoptosis, facilitating cell survival and immune evasion. However, the integrated molecular mechanisms related to EBV miRNAs remain to be investigated. In this review, we summarized the crucial role of EBV miRNAs in epithelial malignancies, especially in EBVaGC. Collectively, EBV miRNAs play a significant role in the viral and host gene regulation network. Understanding the comprehensive potential targets and relevant functions of EBV miRNAs in gastric carcinogenesis might provide better clinical translation.

## Introduction

GC ranks the fourth highest mortality rate worldwide. It is a complex disease with low 5‐year survival rate due to delayed diagnosis. GC can be divided into four molecular groups based on genomic characteristics and clinical features, including chromosomal instability (CIN), genomically stable (GS), microsatellite instability (MSI) and EBV‐associated GC (EBVaGC) 1. EBVaGC is defined by the existence of EBV in the gastric carcinoma cells. It represents about 10% of all GC cases and shows some distinct clinic characters, such as male predominance, relatively younger age and preponderant location in the proximal stomach 2. Molecularly, EBVaGCs display recurrent *PIK3CA* mutations, extreme whole genomic hypermethylation and up‐regulation of JAK2, programmed death receptor‐ligand 1 (PD‐L1) and programmed death receptor‐ligand 2 (PD‐L2) 1. In particular, both MSI subtype and EBVaGCs are associated with better prognosis 3.

miRNAs are small non‐coding, endogenous RNAs containing 18–23 nucleotides that repress targeted gene expression through inducing mRNAs degradation or translational suppression 4, 5. miRNAs recognize and interact with targeted mRNA in the 3′ untranslated region (3′UTR) and inhibit target gene expression 6. Accumulating evidence has suggested that human‐coding miRNAs play crucial roles in cancer initiation and progression 7, 8, 9. Interestingly, EBV is also capable to encode miRNAs by itself and produce a few clusters of miRNAs. EBV‐coding miRNAs regulate both human and EBV transcripts. Up to date, 25 EBV miRNA precursors and 44 mature EBV miRNAs have been identified in miRBase, four of which are encoded from the BamHI fragment H rightward open‐reading frame (BHRF) region and the remainders are from the BamHI‐A region rightward transcript (BART) region 10. Evidence has been emphasizing the cancer‐promoting roles of EBV miRNAs in the carcinogenesis of EBVaGCs. However, the detailed expression and functional roles of EBV miRNAs in the different stages of GC remain unclear. In this review, we focus on recent studies regarding EBV miRNAs in gastric carcinogenesis, particularly on both viral and cellular functional targets of EBV miRNAs. Increasing knowledge of the crucial roles of EBV miRNAs in epithelial malignancies might provide insights on the underlying mechanisms as well as developing novel therapeutic strategies for EBVaGC patients.

## The molecular features of EBVaGC

As EBVaGC is one of the primary epithelial tumours associated with EBV infection, understanding the molecular mechanisms of EBVaGC is critical for early diagnostic and prognostic predictions. Previously, southern blotting results indicated that EBV infection occurred in very initial stages of GC development. By comparing the clonal status of EBV between non‐invasive and early invasive EBVaGC, the latent form of EBV was only detected in non‐invasive carcinoma tissues 11, 12. EBVaGC is involved in latency I neoplasms with specific expression of EBV‐encoded small RNA (EBER), EBV‐encoded nuclear antigens 1 (EBNA1) and non‐transcribed BART RNAs. In human GC cells, latent membrane protein 2A (LMP2A) could also be detected in some EBVaGC cases, while the expression of latent membrane protein (LMP1) is often absent 13, 14.

Not only with viral gene expression, EBVaGC is also characterized with extreme hypermethylation across the whole genome 1. Besides, epigenetic regulation also happens within EBV genome 12. EBV‐associated CpG island methylator phenotype (CIMP) has been proved more extensively and frequently than the other GC subtypes. Furthermore, EBV and MSI methylation patterns are distinct. EBVaGC often demonstrates promoter hypermethylation of a cell cycle‐related gene *CDKN2A*, but lacking *MLH1* hypermethylation 15, 16, 17. Multiple tumour‐suppressive genes are silenced because of the hypermethylation in their promoter regions. As a result, the repression of genes which are expressed in normal cellular activities, such as cell cycle regulation, DNA repair, cell adhesion and metastasis, apoptosis and signal transduction, facilitates gastric carcinogenesis. Using bisulphite sequencing, the expression of DNA methyltransferase 1 (DNMT1) showed elevated expression with a much higher level than the other molecular subtypes 18. Overall, both EBV‐latent infection and unique hypermethylation features may shed lights on early EBVaGC development.

Somatic driver mutations are commonly identified in EBVaGC 19. The mutated genes showed either unaltered or overexpressed in EBV‐positive GC cells, such as *PIK3CA*,* ARID1A* and *BCOR*. The recurrent *PIK3CA* mutations are considered to be the most representative genetic alterations in EBVaGC 1. Based on former evidence, non‐silent PIK3CA mutations presented in nearly 80% of EBVaGC, and 68% cases showed recurrent mutations. Nevertheless, the *PIK3CA* mutations only found in 3–42% of the other GC subtypes and the mutations only existed in the restricted exons in those EBV‐negative GC cases 20, 21. In EBVaGC, *PIK3CA* mutations are spread across many gene segments. Moreover, frequent amplification of *JAK2*,* PD‐L1* and *PD‐L2* were also salient in EBVaGC 22, 23. Signalling pathways associated with the mutations in GCs were also described by TCGA. IL‐12 signalling is prominent in EBV‐positive cells, supporting the role of immune cell signalling. In contrast, RTK‐RAS, RhoA‐ROCK and mitotic network signalling pathways were notable features of the EBV‐negative GC subtypes 1.

Dysregulation of miRNAs has been found contributing to carcinogenesis for more than a decade. MiRNAs that drive carcinogenesis are termed oncomiRs, while those suppress tumour growth are termed anti‐oncomiRs 24. In EBVaGC, on the one hand, cellular miRNAs are regulated by the EBV‐encoded oncogenes. On the other hand, EBV miRNAs regulate the expression of host oncogenes directly 25. Both dysregulated cellular miRNAs and oncogenes therefore enhance cell growth and survival in EBV‐associated cancer 26, 27, 28. Investigating the regulatory mechanisms of miRNAs is no doubt a promising aspect to understand the molecular feature of EBVaGC carcinogenesis.

Generally, tumour cells interact with the tumour microenvironment to promote carcinogenesis 29. Upon such an interaction, EBV host cells could be able to escape from immune surveillance during latency. For instance, extensive infiltration of CD8/CD4‐positive T lymphocytes and overexpression of PD‐L1 augmented immune evasion of cancer cells 30. Disruption of 3′UTR was recently proposed as a unique genetic mechanism contributed to the PD‐L1 up‐regulation 31. Exosomes are membrane‐bound vesicles. To evade from immune reactivity, exosomes are secreted into the microenvironment and transfer viral factors to recipient cells 32. Exosomes harbour a wide range of molecules including miRNAs, which draw the most attention because of their functional roles in carcinogenesis 33. In addition to the gene regulatory role, some exosomal miRNAs were suggested to be Toll‐like receptors (TLRs) ligands and activate immune cells 34. However, the function of exosomal EBV miRNAs has not been fully revealed and the detailed molecular mechanisms are still elusive 33. In 2013, Choi *et al*. found miR‐BART15‐3p was 16‐fold enriched in exosomes and secreted from SUN‐719 cells. Additionally, miR‐BART1‐3p was 12‐fold enriched in the exosomes compared with those in AGS‐EBV cells. However, they did not find significant increased amount of miR‐BART5‐5p in the exosomes. Moreover, their results pointed out that the delivery of miR‐BART15‐3p by exosomes facilitated apoptosis *via* inhibiting BRUCE translation 28. Although the current knowledge about EBV‐encoded miRNAs in EBVaGC is limited, the significant roles of these miRNAs are emerging.

## The EBV and EBV miRNAs

EBV is also called human herpesvirus 4 (HHV‐4), one of the most prevalent viruses in humans 35. According to worldwide statistics, 95% of all adults present serological markers of EBV infection 36, 37. EBV was the first virus identified in human carcinoma in 1964 38. After primary infection, EBV establishes the lifelong virus carrier state, which is called latent infection. In most of cases, the infection is symptomless and harmless, but latent EBV infection may display oncogenic features that is associated with multiple human malignancies originating from epithelial cells, lymphocytes and mesenchymal cells 39. It has been known that EBV infection results in the extensive methylation of both host and viral genome, which promote cellular functions that boost viral persistence and propagation 40.

EBV is an enveloped virus that contains a DNA core surrounded by an icosahedral nucleocapsid and a tegument. The genome of EBV contains a double‐stranded linear DNA in the virus particle, with approximate 172,000 base pairs (bps) and composed of nearly 100 genes 41. The 500‐bp terminal direct repeats and internal repeat sequences part the EBV genome into short and long sequence domains 42.

Up to now, 25 EBV miRNA precursors and 44 mature EBV miRNAs have been identified. The EBV‐associated miRNAs are evolutionarily conserved and differentially regulated 43. In EBV‐latent cells, these miRNAs could be divided into two main clusters, the BHRF1 and BART clusters 44. The BHRF1 cluster expresses four mature miRNAs from three precursor miRNAs. MiR‐BHRF1‐1 is found in the 5′UTR of BHRF1. Its mature sequence is excised from the 5′ arm of the hairpin. MiR‐BHRF1‐2 is found in the 3′UTR of the BHRF1 transcript. The mature sequence of this miRNA is excised from the 3′ arm of the hairpin. The cooperation among BHRF1 miRNAs have been well described in B cells 45, 46. In addition, EBV miRNAs display a cell type‐specific expression pattern. BHRF1 cluster miRNAs are highly expressed in latency Ш and lytic replication‐infected cells, such as B lymphoma cells, but are almost undetectable in cells under latency I and II, such as NPC cells. The BARTs are from multispliced rightward transcripts from the *Bam*H1 A region of the EBV genome. They are responsible for the encoding of other 22 miRNA precursors that produce 44 mature miRNAs in EBV‐infected cells 10. The expression of EBV‐BART miRNAs occurs in all types of latency and reaches extremely high in latency I and II in epithelial hosts. BART miRNAs count a large proportion of the total EBV miRNAs of infected NPC or GC epithelial cells and tissues, suggesting EBV‐BART miRNAs may contribute to the development of epithelial malignancies 44, 47, 48, 49. Maquitz *et al*. previously divided BART miRNAs into subclusters 1 and 2. Subcluster 1 includes miR‐BART1, 3, 4, 15, 5, 6, 16 and 17. Subcluster 2 harbours miR‐BART7, 8, 9, 10, 11, 12, 13, 14, 18, 19, 20 and 21 50. Up to date, several miRNA subtypes were confirmed to be BART cluster 2 members, such as miR‐BARTs 18‐3p, 7‐5p, 10‐3p, 10‐5p, 11‐3p, 13‐5p and 14‐5p, associated with latency type III infection 51, 52, 53. In addition, miR‐BART2‐5p and miR‐BART2‐3p are the downstream members of these two subclusters 24.

Although the knowledge about EBV miRNAs is accumulating, limitations still exist in researches. Firstly, cellular miRNA expression levels may vary in cell lines and clinical primary samples, such as miR‐143 and miR‐155 53. The various levels of cellular miRNA could affect the activity of EBV miRNAs subsequently 50. Secondly, miRNAs exert distinct effects in different cell lines. For example, the regulation of miR‐BART5 to PUMA depends on the PUMA mRNA level in certain cell lines 50. More importantly, due to the absence of an eligible animal model for EBV‐associated carcinogenesis, almost all studies on miR‐BART function are counting on EBV‐infected tumour cell lines. However, some identified miR‐BART targets showed inconsistency in other studies 54. In a recent report, Qiu *et al*. were unable to verify the anti‐apoptotic effect of miR‐BARTs, which has been reported by other groups earlier, in their AGS cell models 55.

## Functional role of EBV miRNAs in EBVaGC

The genetic components of EBV were first discovered in GC by Burke *et al*. in 1990. They detected EBV DNA by polymerase chain reaction (PCR) in a paraffin‐embedded block of an undifferentiated lymphoepithelial gastric carcinoma 56. EBV infection is commonly associated with lymphoid stromal carcinoma, which can be identified by *in situ* hybridization for EBER‐1. EBVaGCs have a better prognosis and lower risk for lymph node metastasis compared to other GC subtypes, which may relate to the prominent lymphoid antitumour response 57. SUN‐719 is a natural originated EBV cell line most similar to EBVaGC cells 58, while the AGS cell line artificially infected by a recombinant EBV was also widely applied 48. Growing discoveries about viral targets and host cellular targets are giving new insights into the EBV miRNA expression profiles and oncogenic roles 26, 59, 60, 61. EBV miRNAs contribute to the initiation and development of EBVaGC by mediating varieties of cellular processes, such as cell proliferation, transformation, apoptosis inhibition and immune evasion. BART miRNAs are expressed at high levels only in EBV‐infected epithelial cancers, but not in EBV‐transformed lymphocytes 62. As BHRF1 miRNAs are barely identified in EBVaGC, BART miRNA cluster comprises all the miRNA functions. The reported BART miRNA functions and targets in EBVaGC are summarized in Table 1. According to recent sequencing data, expression levels of BART miRNAs were consistently high in several kinds of GC cell lines, as well as surgical GC specimens 1. Clarifying the roles of BART miRNA targets and the regulatory pathways is important for the oncogenic mechanisms in EBVaGC.

**Table 1:** Viral and cellular targets of EBV miRNAs in GC

EBV miRNA	Target	Mechanisms
miR‐BART2	Viral DNA polymerase BALF5	Targeting 3′UTR of BALF5 to inhibit transition from latent to lytic viral replication 88
miR‐BART4‐5p	BID	Anti‐apoptosis in GC cell lines 64
miR‐BART5‐5p	PUMA	Inhibit apoptosis and promotes host cell survival in GC cell lines 26
miR‐BART6‐3p	DICER1 LOC553103	Repress miRNA processing 89 Inhibit migration and invasion in GC cell lines 65
miR‐BART9	CDH1	Promote proliferative and invasion activity in SNU719 52
miR‐BART11	FOXP1	Promote inflammation 90
miR‐BART15‐3p	BRUCE	Promote cell apoptosis in AGS‐EBV 28
miR‐BART16	TOMM22	Inhibit cell apoptosis 91
miR‐BART20‐5P	BAD Viral BZLF1 & BRLF1	Inhibit apoptosis and suppresses lytic induction in AGS‐EBV 92, 93 Maintain latency in EBVaGC 69
miR‐BARTs	BIM (BCL2L11)	Inhibit cell death 50.

EBV, Epstein–Barr virus; miRNAs, microRNAs; GC, gastric cancer.

Several studies have profiled EBV‐encoded miRNA expression in EBVaGC tissues and cell lines. EBV‐infected AGS cell line (AGS‐EBV) has been suggested to induce a more transformed phenotype. High levels of BART miRNAs and limited viral protein expression were identified in AGS‐EBV 48. To comprehensively profile the expression pattern of the miRNAs in AGS‐EBV, Marquita *et al*. constructed small‐RNA libraries using Hiseq 2000 sequencing system with uninfected AGS cells and AGS‐EBV in 2013 63. Similar with the NPC cells and other epithelial EBV malignancies, AGS‐EBV cell lines showed elevated BART miRNA expression and the rarely detected amount BHRF1 miRNAs. They also suggested host miRNA expression was changed under the effect on EBV miRNAs. These changes include both a decrease in host tumour suppressor miRNAs and increased viral miRNAs with oncogenic potential 63. In 2015, Shinozaki‐Ushiku *et al*. comprehensively profiled the expression of 44 known EBV miRNAs in tissue samples from patients with EBVaGC. Some EBV miRNAs were found up‐regulated. Based on *in silico* prediction, the targets of these miRNAs were functionally involved in oncogenic pathways such as anti‐apoptosis. Moreover, they performed luciferase assays in EBV‐infected GC cell lines and found a reduction in the pro‐apoptotic protein Bid (the BH3‐interacting domain death agonist), which validated the result. Further, they found miR‐BART4‐5p mimic reduced Bid expression in EBV‐negative cell lines and resulted in apoptosis under serum deprivation 64. In 2017, a profiling of EBV miRNAs in EBVaGC was measured with quantitative PCR. In this report, Tsai *et al*. suggested EBV‐BART4 was the most abundant miRNA, followed by miR‐BART1, miR‐BART2, miR‐BART6, miR‐BART9 and miR‐BART18 in a decreasing order. They also found deletion of miR‐BART9 could elevate E‐cadherin expression as well as miR‐200a and miR‐141 expression, which was accompanied by inhibited proliferation and invasive ability 52. These two profiling studies included all presently known EBV miRNAs from extensive EBV‐infected GC patient samples and selected several high expression miR‐BARTs. Shinozaki‐Ushiku *et al*. discovered the anti‐apoptosis function of miR‐BART4‐5p based on *in silico* target prediction. While Tsai *et al*. mainly concentrated in the clinicopathological features of EBVaGC and shed new lights on the role of EBV miRNAs in malignant transformation through triggering EMT. Collectively, miR‐BARTs show high expression in EBVaGC patients, contributing to the carcinogenesis with unique functional roles. A miRNA can target multiple host and viral genes and inhibit their expression. Cellular targets involved in cell proliferation, transformation and anti‐apoptosis were highly up‐regulated in latent EBV‐infected cell lines, which shown extremely high levels of BART miRNA as well as the associated signalling pathways. P53‐up‐regulated modulator of apoptosis (PUMA) is one of the six members of the BH3‐only group in the Bcl‐2 family, which are essential initiators of apoptosis. MiR‐BART5 targets PUMA by interacting with PUMA mRNA and contributes to the survival of EBV‐infected epithelial cells 26. Bcl‐2‐interacting mediator of cell death (BIM), another BH3‐only group pro‐apoptotic protein, is regulated by miR‐BART4 and miR‐BART5 50. MiR‐BART20‐5p directly inhibited Bcl‐2‐associated death promoter (BAD) to promote EBV‐infected GC cell survival. Translocase of outer mitochondrial membrane 22 homolog (TOMM22) is a receptor for Bcl‐2‐associated B (Bax2), which can be negatively regulated by miR‐BART16. Multiple EBV‐encoded miRNAs involved in EBVaGC by inhibiting N‐myc downstream‐regulated gene 1 (NDRG1). NDRG1 is an oncosuppressor protein and significantly decreased in EBV‐infected epithelial cells. These cellular targets are mainly related to cell apoptosis and tumour suppression. In other words, targeting these mRNAs may cause apoptosis inhibition and cell transformation. Interestingly, not all miR‐BARTs showed oncogenic functions. For example, miR‐BART15‐3p was found promoting apoptosis in AGS GC cells, by targeting the apoptosis inhibitor BRUCE 28. MiR‐BART6‐3p inhibited cell migration and invasion in GC by inhibiting the epithelial–mesenchymal transition (EMT) process through suppressing a long non‐coding RNA (lncRNA) *LOC553103* 65.

In EBVaGC, EBV utilizes its miRNAs not only to regulate host genes but also of the viral functions. Previous studies indicated that both EBV miRNAs and EBV‐mRNAs within the *Bam*H1A region share similar high transcriptional patterns in EBVaGC 1, 66. It is possible that in EBVaGC, EBV‐mRNAs may regulate by EBV miRNAs. By targeting EBV‐mRNAs such as LMP1 (by miR‐BART1, 9, 16 and 17) and LMP2A (by miR‐BART22), BART miRNAs facilitates tumour growth in NPC and lymphoma 55, 59, 67. MiR‐BART2 is thought to target the virally encoded DNA polymerase BALF5 for degradation 68. To date, however, EBV‐mRNA targets by EBV miRNAs were limitedly described in EBVaGC. *BZLF1* and *BRLF1* are early EBV genes triggering lytic replication. miR‐BART20‐5p was suggested to be a key EBV miRNA, directly suppressed the expression of both BZLF1 and BRLF1 to maintain EBVaGC latency. 69.

## Functions of EBV miRNAs in other cancer types

### Lymphoid malignancies

It is known that EBV persistently infects the memory B cell by promoting growth and survival signalling that can contribute to B‐cell lymphomagenesis. The EBV entry mechanisms are different in human B lymphocytes and epithelial cells. The CD21 and human complement receptor type 2 (CR2) on B‐cell surface are responsible for EBV attachment. Burkitt's lymphoma is known to be one of the most prevailing malignancies in equatorial Africa children. There are three different subtypes of Burkitt's lymphoma, including endemic, sporadic and HIV‐associated Burkitt's lymphoma. Almost all endemic Burkitt's lymphoma cases were EBV detectable. Constitutive activation of oncogene c‐Myc is a key pathogenic factor in EBV‐infected B cells, *via* chromosomal translocation, which has been considered as an interaction between EBV and c‐Myc 70, 71. In addition, the finding of EBV terminal DNA repeats was also reported in up to 50% of cases of Hodgkin lymphoma 72, 73. However, the studies of EBV miRNAs in lymphoma are quite limited. The BHRF1 miRNAs inhibited cell apoptosis at the very early stage of primary B‐cell infection and promote cell proliferation. Similarly, the BART miRNAs were suggested to prevent apoptosis in Burkitt's lymphomas by inhibiting caspase‐3 and to enhance cell growth of primary infected B cells 26, 50, 74.

### Npc

EBV DNA copies can be detected in nasopharynx lesions 75 and invasive NPC 76. Although lots of people carrying EBV in China only develop mononucleosis, the viral products of EBV may potentially contribute to the high aggressiveness of NPC under certain circumstances 75, 77, 78. In NPC, latency was classified as type II and several EBV miRNAs participated to regulate both cellular gene expression and EBV gene expression, supporting host cell survival and immune escape 26, 67, 79. BHRF1‐encoded miRNAs are undetectable in NPC tissues and cell lines 49, 80. BART miRNAs were found highly expressed in NPC. Many of the cellular targets of BART miRNAs are tumour suppressors, suggesting the oncogenic role of BART miRNA in the progress of NPC. MiR‐BARTs affect multiple cellular functions including cell proliferation, survival and evasion of host immune response. Similar to EBVaGC, host targets of BART‐encoded miRNAs in NPC include PUMA, Bim, as well as TOMM22 26, 50, 81. Besides these tumour suppressor genes, BART miRNAs target putative immune regulatory molecules like importin 7 (IPO7), Dicer and major histocompatibility complex class I‐related chain B (MICB) 82. In addition, the importance of miR‐BART‐7 has already been addressed in NPC metastasis by regulating the PTEN‐PI3K/Akt‐EMT pathway 83. Moreover, miR‐BART1 inducing tumour metastasis by regulating PTEN‐dependent pathway has been identified 84. EBV‐encoded gene LMP1 was targeted by miR‐BART1, miR‐BART9, miR‐BART16 and miR‐BART17 59, and LMP2A was a target of miR‐BART22 67. Importantly, BART miRNAs present in the serum of NPC patients and might be used as biomarkers 85. MiR‐BART1‐5p measurement could act as an efficient method assisting clinical diagnosis of NPC 86. More recently, Yoshizaki *et al*. estimated the circulating miR‐BART2‐5p, miR‐BART17‐5p and miR‐BART18‐5p copy number and found miR‐BART17‐5p could serve as a post‐treatment biomarker for prediction of recurrence in EBV‐related NPCs 87.

## Conclusion and future perspectives

The aberrant expression of EBV miRNAs plays important roles in multiple epithelial malignancies including EBVaGC. The members in BART miRNA family exclusively accelerate the malignant progress by targeting viral or host cellular genes. Collectively, EBV‐encoded miRNAs may cooperate and exert fundamental influence on maintaining latency, cell growth and immune evasion in EBVaGC. Although the expression patterns and functional roles of BART miRNAs have been revealed, the integrate mechanisms of BART miRNA in gastric carcinogenesis are not yet precisely and fully known. Several issues need to be addressed in the future studies. Firstly, the EBV miRNA expression spectrum needs to be confirmed for screening the most up‐regulated and promising miRNAs in EBVaGC. Secondly, the comprehensive revealing of the host cellular targets of the EBV miRNA might help to understand the whole regulatory network and identify novel therapeutic targets for GC.

## Conflict of interest

The authors declare no conflict of interest.
